# Therapeutic effects of different doses of polyethylene glycosylated porcine glucagon-like peptide-2 on ulcerative colitis in male rats

**DOI:** 10.1186/s12876-017-0593-x

**Published:** 2017-03-04

**Authors:** Ke-ke Qi, Jia-jia Lv, Jie Wu, Zi-wei Xu

**Affiliations:** Institute of Animal Science, Zhejiang Academy of Agricultural Sciences, 198 Shiqiao Road, Jianggan, Hangzhou, 310021 China

**Keywords:** Porcine glucagon-like peptide-2, Ulcerative colitis, Tight junction, Inflammation cytokines

## Abstract

**Background:**

Polyethylene glycosylated (PEGylated) porcine glucagon-like peptide-2 (pGLP-2) considerably increases half-life and stability compared with the native pGLP-2, but the effective dose for intestinal damage is still unclear. This study aims to evaluate the available dose of polyethylene glycosylated porcine glucagon-like peptide-2 (PEG-pGLP-2), a modified, long-acting form of pGLP-2 in an experimental rat model of ulcerative colitis.

**Methods:**

Thirty-five male rats were randomly assigned into five groups: control, dextran sodium sulphate (DSS), DSS + PEG–pGLP-2(L), DSS + PEG–pGLP-2(M) and DSS + PEG–pGLP-2(H). Rats in control group received only water; other rats were fed with 5% (w/v) DSS and intraperitoneally administered with 12.5, 25 and 100 nmol/kg PEG–pGLP-2 daily for 6 days.

**Results:**

Compared with the control treatment, DSS treatment significantly (*p* < 0.05) decreased body weight change, colonic length, duodenal villus height and expression of zonula occludens-1, whereas significantly (*p* < 0.05) increased colonic damage score and expression of claudin-1, interleukin (IL)-1, IL-7, IL-10, interferon-γ and tumour necrosis factor (TNF)-α in colon. However, the three doses of PEG–pGLP-2 all reduced these effects; these treatments significantly (*p* < 0.05) increased body weight change and duodenal villus height, whereas significantly (*p* < 0.05) decreased colonic damage score and expression of IL-1, IL-7 and TNF-α in colon. Specifically, low-dose (12.5 nmol/kg/d) PEG–pGLP-2 was effective.

**Conclusions:**

These results indicated that PEG–pGLP-2 is a novel and potentially effective therapy for intestinal healing in a relatively low dose.

**Electronic supplementary material:**

The online version of this article (doi:10.1186/s12876-017-0593-x) contains supplementary material, which is available to authorized users.

## Background

Frequent injection of porcine glucagon-like peptide-2 (pGLP-2) because of the rapid degradation of pGLP-2 by the enzyme dipeptidyl peptidase-IV (DPP-IV) is the main impediment of pGLP-2 as a potential therapeutic agent for intestinal dysfunction and damage in weaning piglets [1]. For the first time, our team designed and purified polyethylene glycosylated (PEGylated) pGLP-2 (PEG-pGLP-2), whose half-life (t_1/2_) is 16-fold longer than that of pGLP-2 in DPP-IV in vitro [2]. PEG-pGLP-2 infusion could alleviate the severity of intestinal injury in weaning piglets [3, 4]. The t_1/2_ of teduglutide, a GLP-2 analogue, which is a candidate for the treatment of short bowel syndrome (SBS) in two phase III clinical studies [5], is 2.99 h in male patient [6]. Recently, our team proved that the t_1/2_ of PEG-pGLP-2 is 3.38 h in male rats [7]. These findings provide a new perspective to the potential therapeutic use of PEG-pGLP-2 for weaning and diarrhoeal diseases in young pigs.

However, no reports about the therapeutic effect of PEG-pGLP-2, except that of our team, are available. Little information is also known about the dose and frequency of administration with PEG-pGLP-2. Thymann et al. [8] found that injection of acylated GLP-2 analogue 25 μg/(kg BW · 12 h) for 5 days increases intestinal weight and activity of brush border enzymes in weaned pigs when only using long-acting analogues or under diarrhoeal conditions. Kaji et al. [9] demonstrated high doses (240 μg/kg/day for 5 days) of continuous infusion result in the largest increases in microscopic and gross intestinal morphologic parameters in parenterally maintained rats. Similarly, Nakame et al. [10] found that high dose of GLP-2 (800 μg/kg BW every 6 h before stress) administration improves the incidence and survival rate of necrotising enterocolitis (NEC) in rats. Nevertheless, Sigalet et al. [11] found the effects of subcutaneous injection of weaning pigs with GLP-2 at a dose of 40 μg/kg/day for 42 days are limited to the gastrointestinal tract; GLP-2 could increase crypt cell proliferation rates and decrease in apoptosis; no effects were observed on activity, appetite, behaviour or food intake. Deng et al. [12] found that both low and high doses of exogenous GLP-2 (2 or 10 nmol/kg BW per day for 7 consecutive days) improve the growth of weaned piglets and protect them against LPS-induced intestinal damage. The various results of therapeutic methods of GLP-2 may be caused by the different agents, experimental animals and test environments. However, the substantially long t_1/2_ of PEG-pGLP-2 provides us confidence to utilise it for post-weaning diarrhoea syndrome.

The present study was designed to examine the effect of different doses of long-acting pGLP-2, that is, PEG-p[Gly^2^]GLP-2, on growth performance, intestinal morphology and expression levels of tight junction (TJ) proteins and inflammatory cytokines in an experimental rat model of ulcerative colitis. Results of this study may provide the dose reference for application of PEG-p[Gly^2^]GLP-2 in post-weaning diarrhoea syndrome.

## Methods

### Materials

Porcine [Gly^2^] GLP-2 (HGDGSFSDEMNTVLDNLATRDFINWLLHTKITDSL, > 98%) was synthesised by Chinese Peptide Company (Hangzhou, China). Monomethoxy PEG–succinimidyl propionate (mPEG–SPA; molecular weight (MW) = 5 kDa) was purchased from Beijing Kaizheng Biotech Development Co., Ltd. (Beijing, China). The ion-exchange chromatography (IEC) resin and column used were CM Sepharose Fast Flow (GE Healthcare Bio-Science AB, Uppsala, Sweden). Ultrafiltration membranes with a MW cutoff of 3000 were purchased from Millipore Corporation (Billerica, MA, USA). Dextran sodium sulphate (DSS) (MW = 36 000–50 000) was purchased from MP Biomedicals China (Shanghai, China). RNAiso Plus, PrimeScript®RT-reagent with gDNA Eraser Kit and SYBR® Premix Ex TaqTM were purchased from Takara Biotechnology Co., Ltd. (Dalian, China). RNA locker was purchased from TIANDZ (Beijing, China). Unless otherwise specified, all other chemicals and reagents were of analytical grade from Sigma–Aldrich and Fluka (Milan, Italy).

### Preparation of PEG-p[Gly^2^]GLP-2

The mono-PEGylated p[Gly^2^]GLP-2 conjugates were prepared and purified as previously described [3]. Briefly, 1 mg/mL of p[Gly^2^]GLP-2 in 50 mmol/L Tris-HCl buffer solution was reacted with 4 mole excess of mPEG_5k_-SPA at room temperature for 30 min. The reaction was quenched by 1% trifluoroacetic acid/deionised water. The PEGylated p[Gly^2^]GLP-2 mixtures were separated with IEC with C M Sepharose Fast Flow resin. The equilibration phase was 20 mmol/L of acetate buffer (pH 4.0), and the elution phase was 20 mmol/L acetate buffer (pH 4.0) + 1 mol/L NaCl. The PEGylated p[Gly^2^]GLP-2 products were eluted in a linear gradient ranging from 0 to 60% for 60 min and to 100% for 30 min with 1 mL/min of elution buffer at 215 nm. The PEGylated products were collected, desalted, concentrated through 3 kDa cutoff, ultrafitrated and lyophilised to powder before the animal study.

### Animal ethics statement and experimental protocol

Animal studies were conducted in accordance with the guidelines of the Zhejiang Farm Animal Welfare Council of China and approved by the ethics committee of Zhejiang Academy of Agricultural Sciences. Thirty-five male Sprague–Dawley rats were allocated into five treatments: control, DSS, DSS + PEG–pGLP-2(L), DSS + PEG–pGLP-2(M) and DSS + PEG–pGLP-2(H). Each treatment was performed in seven replicates on one rat. The rats were housed in a plastic bottom, wire-lid cages, maintained on a 12 h: 12 h light–dark cycle and allowed to acclimatise to the animal facility for 3 days. Rats in control group received only water; other rats were fed with 5% (w/v) DSS through their drinking water for 6 days from the first day of experiment, followed by 2 days of water consumption. The rats from the DSS + PEG–pGLP-2(L), DSS + PEG–pGLP-2(M) and DSS + PEG–pGLP-2(H) groups were intraperitoneally administered with 12.5, 25 and 100 nmol/kg PEG–pGLP-2, respectively, in 0.3 mL of saline solution daily from day 1 to day 6 of the trial. The rats in the control and DSS groups were administered with the same volume of saline solution. During the experimental period, behaviour and body weight were monitored daily. On day 8, all rats were anesthetised and sacrificed to collect their intestinal sample.

### Collection samples

Samples collection was prepared as previously described [4]. The segment from the pylorus to the Trietz ligament and from the ileocecal valve to the rectum was considered the duodenum and colon, respectively; these segments were cut free from their mesentery and immediately placed on ice. The duodenum and colon were cleaned of luminal contents, weighed and measured with a balance and a ruler. The middle segments (1 cm) of duodenum and colon were fixed in 10% formalin for 48 h and embedded in paraffin using standard techniques for histology. One centimetre of the distal segment of colon was collected for RNA isolation, which was immediately placed in 1 mL of RNA locker.

### Histology

Histology was prepared as previously described [3]. The paraffin-embedded sections were cut at 4 μm thickness and stained with haematoxylin and eosin. Duodenum villus-plus-crypt height was imaged using an Olympus DP-71 digital camera, and quantification was performed using an image processing system. Approximately 12 longitudinally oriented villi were measured from each H&E-stained section to make *n* = 1. All measurements were performed in a blinded manner. Colonic damage score (CDS), was evaluated in a blinded manner in tissue sections based on a grade scale ranging from 0 to 3 to measure the amount and depth of inflammation. The amount of crypt damage or regeneration was measured in a scale of 0–4, as indicated in Dieleman et al. [13]. These changes were also quantified in terms of the percentage involvement of the disease process: (1) 1–25%, (2) 26–50%, (3) 51–75% and (4) 76–100%. Each section was separately scored for each feature by establishing the product of the grade for that feature and the percentage involvement. The sum of the products of the four scores was the CDS for each rat.

### Real-time PCR

Total RNA was isolated using RNAiso Plus kits following the manufacturers’ instructions [4]. A 2 μL aliquot of the total RNA was used for first-strand cDNA synthesis using PrimeScript® RT-reagent Kit with gDNA Eraser. Real-time PCR was carried out with ABI plus one (Life Technologies, Carlsbad, CA, USA) using SYBR Premix Ex TaqTM. The primers used were in Table 1. RT-PCR analysis was performed with an ABI Step One Plus Real-Time PCR System (Applied Biosystems, Inc.) using SYBR® Premix Ex TaqTM. PCR reactions were performed in triplicate under the following conditions: 95 °C for 30 s; 40 cycles of 95 °C for 5 s and 60 °C for 30 s. Fluorescent data for quantification were collected at the end of each cycle. Melting curve analysis was performed at 55–95 °C to verify the single product generation at the end of the assay. Standard curves were generated based on the data obtained from the standards of the 2–2^−6^ dilution series template. Standard curve analysis was performed by using Step One Software (v2.2, Applied Biosystems, Inc.). Data were normalised to GADPH using the 2^−△△Ct^ quantitation method.

**Table 1:** Primer sequences (5′ to 3′) used for the quantitative polymerase chain reaction

Gene	Forward primer	Reverse primer
ZO-1	CCATCTTTGGACCGATTGCTG	TAATGCCCGAGCTCCGATG
Occludin	ACGGTGCCATAGAATGAGATGTTG	CAGCTAGTTGTTCATTTCTGCACCA
Claudin-1	CATCGCAGCTACTTGCCAGT	TTTTTTTTTTTTTTTTGCAAAAACGA
IL-1	TCAGGAAGGCAGTGTCACTCATTG	ACACACTAGCAGGTCGTCATCATC
IL-6	TAGAGTCACAGAAGGAGTGG	GCCAGTTCTTCGTAGAGA
IL-7	AACCTCCAAGAAACTACTGCC	CACCGATGCATGGTTGCTAAC
IL-10	CTCAGCACTGCTATGCTGCCTGCT	TCTTCACCTGCTCCACTGCCTTGC
INF-γ	TGTCATCGAATCGCACCT	TCAGCACCGACTCCTTTT
TNF-α	ACCCCCAACCTATGAAGAAA	TCCACGCAAAACGGAATGAA
GADPH	CCGAGGGCCCACTAAAGG	GCTGTTGAAGTCACAGGAGACAA

### Statistical analysis

Data are expressed as the mean ± SD. Statistical differences between groups were compared through ANOVA with Duncan’s multiple comparison tests at an alpha value of 0.05.

## Results

### Growth performance

As shown in Table 2, compared with the control treatment, the rats treated with 5% DSS for 6 days exhibited significantly decreased body weight change and colonic length (*p* < 0.05). Compared with the DSS group, body weight change and ratio between colonic weight and body weight of rats in DSS + PEG–pGLP-2(L) and DSS + PEG–pGLP-2(M) groups significantly (*p* < 0.05) increased, and the colonic length of rats in DSS + PEG–pGLP-2(L) group significantly (*p* < 0.05) increased; the body weight change in DSS + PEG–pGLP-2(H) also significantly (*p* < 0.05) increased. No significant differences in intestinal length and ratio of intestinal weight/body weight were observed among the five groups.

**Table 2:** Effects of polyethylene glycosylated (PEGylated) porcine glucagon-like peptide-2 (pGLP-2) on growth performance of colitis rats

Groups	Body weight change, %	Intestinal length, cm	Colonic length, cm	Intestinal weight (mg)/body weight (g)	Colonic weight (mg)/body weight (g)
Control	30.48 ± 3.44^a^	94.72 ± 4.93	15.69 ± 1.90^a^	30.61 ± 2.22	11.19 ± 1.90^b^
Dextran sodium sulphate (DSS)	11.90 ± 5.04^c^	92.88 ± 9.78	12.40 ± 1.94^b^	35.19 ± 1.22	9.15 ± 1.79^b^
DSS + PEG–pGLP-2(L)	21.45 ± 2.00^b^	94.50 ± 7.07	14.74 ± 1.90^a^	34.51 ± 3.09	14.13 ± 2.77^a^
DSS + PEG–pGLP-2(M)	21.46 ± 4.65^b^	99.10 ± 8.03	14.48 ± 1.42^ab^	38.91 ± 4.23	14.22 ± 0.38^a^
DSS + PEG–pGLP-2(H)	20.63 ± 3.4^b^	99.56 ± 6.82	13.42 ± 0.80^ab^	35.87 ± 1.73	11.52 ± 0.64^b^

Data are expressed as means ± SD; no significant difference exists in that with no letter or the same letters (*p* > 0.05), and different letters (*p* < 0.05) indicate statistically significant difference

### CDS of colon

**Fig. 1:**
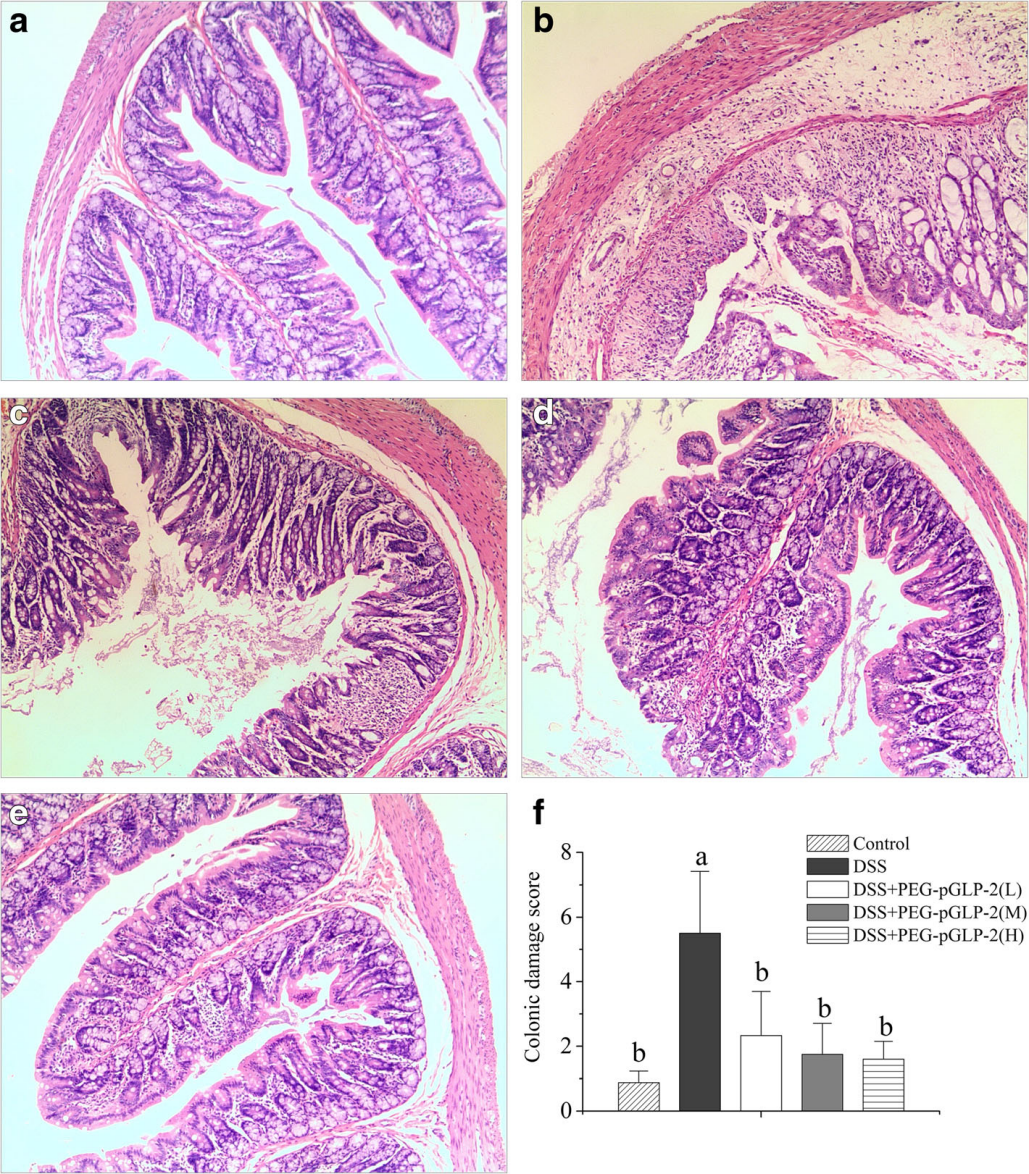
Effects of PEG-pGLP-2 on the morphology and inflammation score of colon. **a**, **b**, **c**, **d**, **e** is the morphology of colon in group of Control, DSS, DSS + PEG–pGLP-2(L), DSS + PEG–pGLP-2(M), DSS + PEG–pGLP-2(H). Data of the colonic inflammation score (**f**) are expressed as means ± SD and different letters (*p* < 0.05) indicate statistically significant difference

The colonic morphology and CDS of the rats from the five treatments are shown in Fig. 1a–f. Compared with the control treatment, the CDS (Fig. 1f) of colon in rats of DSS group significantly (*p* < 0.05) increased, which was characterised as induced oedema of the intestinal wall, mucous epithelium destruction and multifocal dropouts of entire crypts in a large part of the colon (Fig. 1b). Compared with DSS group, the CDS (Fig. 1f) of colon in rats of DSS + PEG–pGLP-2(L) (Fig. 1c), DSS + PEG–pGLP-2(M) (Fig. 1d) and DSS + PEG–pGLP-2(H) (Fig. 1e) groups significantly (*p* < 0.05) decreased; such change was characterised as slight oedema of the intestinal wall, reconstruction of the mucous epithelium, increased goblet cells and decreased multifocal dropouts and focal lesions inflammatory cells; particularly, the colon morphological structures of DSS + PEG–pGLP-2(M) (Fig. 1d) and DSS + PEG–pGLP-2(H) (Fig. 1e) group were almost normal with no damage.

### Morphology of duodenum

As shown in Table 3, compared with the control treatment, the villi height of duodenum in rats of DSS group significantly (*p* < 0.05) decreased. Compared with DSS group, the villi height of duodenum in rats of DSS + PEG–pGLP-2(L), DSS + PEG–pGLP-2(M) and DSS + PEG–pGLP-2(H) groups significantly (*p* < 0.05) increased, and the ratio of villi height/crypt depth in rats of DSS + PEG–pGLP-2(M) group significantly (*p* < 0.05) increased. No significant differences in crypt depth of duodenum in rats were observed among the five groups.

**Table 3:** Effects of PEG-pGLP-2 on morphology of duodenum in colitis rats

Groups	Villus height, μm	Crypt depth, μm	Villus height/Crypt depth
Control	1016.37 ± 49.60^a^	401.79 ± 57.00	2.22 ± 0.12^b^
DSS	777.99 ± 104.34^b^	337.15 ± 30.19	2.28 ± 0.16^b^
DSS + PEG–pGLP-2(L)	981.46 ± 136.04^a^	400.76 ± 62.04	2.66 ± 0.49^ab^
DSS + PEG–pGLP-2(M)	1006.42 ± 98.11^a^	357.43 ± 52.21	2.98 ± 0.49^a^
DSS + PEG–pGLP-2(H)	1003.03 ± 96.41^a^	419.63 ± 51.00	2.39 ± 0.30^b^

Data are expressed as means ± SD; no significant difference exists in that with no letter or the same letters (*p* > 0.05), and different letters (*p* < 0.05) indicate statistically significant difference

### Expression of TJ and inflammation cytokines in colon

**Fig. 2:**
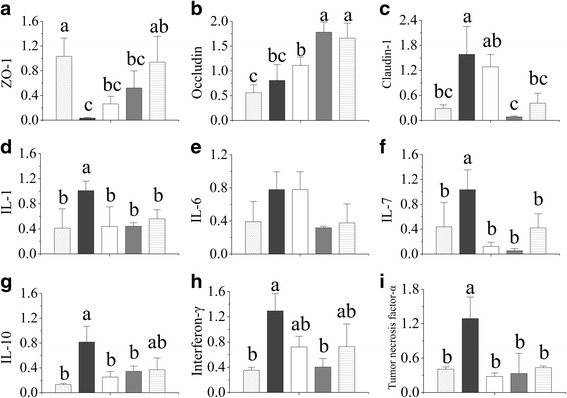
Effects of PEG-pGLP-2 on the expression levels of tight junction proteins and inflammatory cytokines in colon of colitis rats. **a**, **b**, **c** is the expression levels of ZO-1, Occludin and Claudin-1. **d**, **e**, **f**, **g**, **h**, **i** is the expression levels of IL-1, IL-6, IL-7, IL-10, IFN-γ andTNF-α. Data are expressed as means ± SD; no significant difference exists in that with no letter or the same letters (*p* > 0.05), and different letters (*p* < 0.05) indicate statistically significant difference

The expression levels of TJ proteins in colon are shown in Fig. 2a–c. Compared with the control treatment, the expression of zonula occludens-1(ZO-1) (Fig. 2a) in rats of DSS group significantly (*p* < 0.05) decreased, whereas the expression of claudin-1 (Fig. 2c) in rats of DSS group significantly (*p* < 0.05) increased. Compared with DSS group, the expression of ZO-1 (Fig. 2a) in rats of DSS + PEG–pGLP-2(H) and occludin (Fig. 2b) in rats of DSS + PEG–pGLP-2(M) and DSS + PEG–pGLP-2(H) groups significantly (*p* < 0.05) increased; moreover, the expression of claudin-1 (Fig. 2c) in rats of DSS + PEG–pGLP-2(M) and DSS + PEG–pGLP-2(H) group significantly (*p* < 0.05) decreased.

The expression levels of inflammatory cytokines in colon are shown in Fig. 2d–i. Compared with the control treatment, the expression of interleukin (IL)-1 (Fig. 2d), IL-7 (Fig. 2f), IL-10 (Fig. 2g), interferon-γ (IFN-γ) (Fig. 2h) and tumour necrosis factor-α (TNF-α) (Fig. 2i) in rats of DSS group significantly (*p* < 0.05) increased. Compared with DSS group, the expression of IL-1 (Fig. 2d), IL-7 (Fig. 2f) and TNF-α (Fig. 2i) in rats of DSS + PEG–pGLP-2(L), DSS + PEG–pGLP-2(M) and DSS + PEG–pGLP-2(H) groups significantly (*p* < 0.05) decreased; the expression of IL-10 (Fig. 2g) in rats of DSS + PEG–pGLP-2(L) and DSS + PEG–pGLP-2(M) groups significantly (*p* < 0.05) decreased; the expression of IFN-γ (Fig. 2h) in rats of DSS + PEG–pGLP-2(M) group significantly (*p* < 0.05) decreased. No significant differences in the expression of IL-6 (Fig. 2e) in colon of rats were observed among the five groups.

## Discussion

This study is the first about the therapeutic effects of different doses of PEGylated pGLP-2 on DSS-induced colitis in rats. In this experiment, administration of 12.5, 25 or 100 nmol/kg/d PEG–pGLP-2 for 6 days could reduce the severity of colitis, improve the body weight and colonic weight, and decrease the production of inflammatory cytokines. PEG–p[Gly2]GLP-2 at 12.5 nmol/kg/d for 6 days is effective for recovering the colonic damage by DSS, which is indicated by colonic weight, length, damage score and expression of inflammatory cytokines (IL-1, IL-7, IL-10 and TNF-α).

DSS-induced ulcerative colitis is a reproducible model with symptoms of weight loss and colon shortening. GLP-2 can prevent DSS damage in mice, reverse body weight loss, decrease colonic inflammation and increase colonic integrity [14, 15], and the PEG-pGLP-2 here showed similar effects. There were some intreasting results in this study. First, PEG-pGLP-2 reduced the expression of both inflammation cytokines (IL-1, IL-7, TNF-α) and anti-inflammation cytokines (IL-10). Which was similar to previous reports regarding dextran sulphate-induced colitis [14, 16] and weaning piglets challenged with LPS [4]. Ivory et al. [17] demonstrated the anti-inflammation effects of GLP-2 are IL-10 independent in IL-10 knockout mouse. The anti-inflammatory effects of GLP-2 were assumed to be associated with the increased crypt proliferation and decreased crypt apoptosis, which presumably aid healing of lesions and subsequently reduce the production of inflammatory cytokines [18]. Second, expression of tight junction protein ZO-1 and Occludin increased while the tight junction protein Claudin-1 decreased. Tight junctions is an important junctional complex in epithelial cells, and contains several unique proteins, namely, claudins, the transmembrane protein occludin and ZOs [19]. The impact of GLP-2 on the TJ proteins (ZO-1 and occludin) of the jejunum epithelia was confirmed by immunofluorescence staining in ob/ob mice injected with GLP-2 [20]. The different changes of Claudin-1 in tight junction protein could be explained by a possible compensatory role for claudin-1 in the damaged TJ of Poritz et al. [21], which found in dextran sulfate sodium (DSS) model of colonic inflammation, a decrease in ZO-1 but also a counterintuitive increase in claudin-1.

Effects of exogenous GLP-2 or its long-acting analogues on intestinal growth and healing have been studied in different animal models. Early time, the effective dose of GLP-2 is considerably high; Kato et al. [22] demonstrated that GLP-2 can enhance normal rat small intestine mucosal mass and absorption at 50 mg/kg/d for 14 days. The findings of Thymann et al. [8] may explain the above result; they found that animal living environment (normal animal or with disease) and modified GLP-2 are important for the intestinal therapeutic effects for piglets. Thymann et al. [8] also found that the significant effects of GLP-2 improve gut structure and function in weanling pigs only under diarrhoeal conditions and when using long-acting GLP-2 analogues. Thus, the dose of native GLP-2 shows relatively high effect. Nakame et al. [10] found subcutaneous administration of 800 μg/kg/day (224 nmol/kg/d) GLP-2 for 4 days could reduce the severity of NEC and improve the survival rate in rats. In the study of Kaji et al. [9], the continuous infusion of 240 μg/kg/day (67 nmol/kg/d) GLP-2 for 5 days induces intestinal growth and increases weight gain using a total parenteral nutrition (TPN) supported model in the rat. Thymann et al. [8] found that 200 μg/ kg · 12 h (56 nmol/kg · 12 h) GLP-2 for 7 days only increases goblet cell density, but shows limited effects on diarrhoea. No differences exist between two regimens (continuous GLP-2 infusion versus three daily GLP-2 injections) of 1 mg/day GLP-2 for 21 days on intestinal absorption in SBS patients [23]. Infusion of 100 μg/kg/d GLP-2 for 7 days augments adaptive growth and digestive capacity of the residual small intestine in a rat model of mid–small bowel resection [24]. Injection duration is also a major factor influencing the therapeutic effects. Sigalet et al. showed that chronic treatment of weaning pigs with GLP-2 at 40 μg/kg/day given by s.c. injection twice daily for 42 days increases the overall villus height and crypt depth; no effects were observed on activity, appetite, behaviour or food intake in weaning pigs [11].

However, when the peptide chain is changed or modified, the effective dose of GLP-2 is considerably low. L’Heureux and Brubaker (2003) used 40 μg/kg · q12 h (11.5 nmol/kg · 12 h) human [Gly^2^] GLP-2 for 10 consecutive days, which increases survival and small intestinal weight, and decreases body weight loss and colonic damage in DSS-mice [18]. Thymann et al. [8] found that 25 μg/ kg · 12 h of a long-acting acylated GLP-2 analogue for 5 days increases intestinal weight and activity of brush border enzymes in a low-sanitary environment. Pretreatment with a long-acting DPPIV inhibitor K579 (1 mg/kg, ig) for 1 day prevents the formation and promotes healing of IND-induced intestinal ulcers, although high-dose K579 (3 mg/kg) reverses the preventive effect [25]. Teduglutide, a DPP-IV-resistant GLP-2 analogue given subcutaneously for 21 days at three dose levels (30, 100 and 150 μg/kg/day), significantly increases intestinal wet weight absorption in SBS patients [26]. In the study of a neonatal piglet jejunostomy model by Thymann et al. [27], a dose-dependent (0.01, 0.02, 0.1 or 0.2 mg/kg/day teduglutide) increase in weight per length of the remnant intestine is obtained, but functional and structural endpoints, including digestive enzyme activity and enteral nutrient absorption, are not affected by treatments. XTEN is a long, unstructured, non-repetitive, hydrophilic sequence of amino acids. With the fusion of XTEN to the C-terminus of GLP2-2G, the serum half-life of GLP2-2G-XTEN in rats is 38 h, and 25 nmol/kg/day GLP2-2G-XTEN for 12 days significantly increases the length, reduces the number of trans-ulcerations and adhesions, and reduces the TNF-α content of the small intestine in rat indomethacin-induced disease model [28].

PEGylated pGLP-2, a site-specific mono-PEGylated derivative, was designed and purified by our team. This derivative exhibits longer half-life than native pGLP-2 in vitro and can reduce the severity of colonic injury in murine colitis [2]. We demonstrated that 10 nmol/kg/d PEG–pGLP-2 infusion alleviates the severity of intestinal injury in weaning piglets challenged with LPS by reducing the secretion of inflammatory cytokines and increasing the villus height/crypt depth ratio [3]. To provide references for the intestinal injury of weaned pigs by PEG–p[Gly^2^]GLP-2, the pharmacokinetics of PEG–p[Gly^2^]GLP-2 was conducted. The results showed that the half-life of p[Gly^2^]GLP-2 and PEG–p[Gly^2^]GLP-2 is 49 and 202 min in rat, respectively, which is longer than the half-life of intact pGLP-2 (8.4 min) [1]. PEGylated p[Gly^2^]GLP-2 significantly improves the pharmacological profiles; increases half-time, peak time and mean residence time; decreases reduce clearance rate; and improves bioavailability [7]. Lee et al. [29] used a similar methodology to project the half-life of the GLP-1fusion; PEG(2 k)-Lys-GLP-1 improves the half-life with a 16- and 3.2-fold increase for intravenous and subcutaneous administrations, respectively.

The present study mainly aims to provide the dose reference for application of PEG-pGLP-2 in weaned pig. The effective dose of GLP-2 in pig is relatively low. Both low (2 nmol/kg BW/day) or high (10 nmol/kg BW/day) dose of human [Gly2]GLP-2_1–34_ 2 for 7 days improves the growth of weaned piglets and protects them against LPS-induced intestinal damage [12]. TPN-fed neonatal piglets with infused iv at three rates (2.5, 5.0 and 10.0 nmol/kg/d) of GLP-2 for 7 days exhibit increased small intestinal weight, DNA and protein content, and villus height [30]. This observation may be explained by the long half-life in different animals. Alters et al. [28] demonstrated that the serum half-life of GLP2-2G-XTEN in mice, rats and monkeys is 34, 38 and 120 h, respectively. Therefore, the longer half-life of PEG–p[Gly2]GLP-2 implies a lower total dose requirement in weaned piglet.

## Conclusions

Our results demonstrated three doses (12.5, 25 or 100 nmol/kg/d) of PEG–pGLP-2, a long-acting pGLP-2, reduces the ulcerative colitis in male rats by recovering the body weight, colonic length, colonic damage score and production of inflammatory cytokines. PEG–pGLP-2 is a novel and potentially effective therapy for intestinal healing in a relatively low molar dose (12.5 nmol/kg/d).

## Additional file

Additional file 1: Raw data of growth performance, morphology of duodenum, colonic damage score, CT of tight junction proteins and inflammatory cytokines in colon. (XLSX 113 kb)

## Abbreviations

CDS: Colonic damage score
DPP-IV: Dipeptidyl peptidase-IV
DSS: Dextran sodium sulphate
IL: Interleukin
PEG–pGLP-2: Polyethylene glycosylated porcine glucagon-like peptide-2
t_1/2_: Half-life
TJ: Tight junction
ZO-1: Zonula occludens-1
